# New Foil

**Published:** 1871-01

**Authors:** 


### NEW FOIL.

We have been using a foil just introduced, bearing the name of Geo. Watt & Co. as manufacturers, better than which we have never used. It is soft and pliable, yet very adhesive. It seems to have about every quality that can be expected in gold foil. We advise all to try it. So far as we have tested it it is very uniform in working quality. T.

